# Structural insights into context-dependent inhibitory mechanisms of chloramphenicol in cells

**DOI:** 10.1038/s41594-024-01441-0

**Published:** 2024-12-12

**Authors:** Liang Xue, Christian M. T. Spahn, Magdalena Schacherl, Julia Mahamid

**Affiliations:** 1 Structural and Computational Biology Unit, European Molecular Biology Laboratory (EMBL), Heidelberg, Germany; 2 Institut für Medizinische Physik und Biophysik, Charité–Universitätsmedizin Berlin, corporate member of Freie Universität Berlin and Humboldt-Universität zu Berlin, Berlin, Germany; 3 Cell Biology and Biophysics Unit, European Molecular Biology Laboratory (EMBL), Heidelberg, Germany; 4 Present Address: Key Laboratory of Biomacromolecules, Institute of Biophysics, Chinese Academy of Sciences, Beijing, China

**Keywords:** Cellular microbiology, Molecular biophysics, Cryoelectron tomography, Ribosome

## Abstract

Ribosome-targeting antibiotics represent an important class of antimicrobial drugs. Chloramphenicol (Cm) is a well-studied ribosomal peptidyl transferase center (PTC) binder and growing evidence suggests that its inhibitory action depends on the sequence of the nascent peptide. How such selective inhibition on the molecular scale manifests on the cellular level remains unclear. Here, we use cryo-electron tomography to analyze the impact of Cm inside the bacterium *Mycoplasma pneumoniae*. By resolving the Cm-bound ribosomes to 3.0 Å, we elucidate Cm’s coordination with natural nascent peptides and transfer RNAs in the PTC. We find that Cm leads to the accumulation of a number of translation elongation states, indicating ongoing futile accommodation cycles, and to extensive ribosome collisions. We, thus, suggest that, beyond its direct inhibition of protein synthesis, the action of Cm may involve the activation of cellular stress responses. This work exemplifies how in-cell structural biology can expand the understanding of mechanisms of action for extensively studied antibiotics.

## Abstract (web summary)

Xue et al. visualize how the antibiotic chloramphenicol reshapes the translation landscape and induces ribosome collision in bacterial cells, illuminating its context-dependent action across atomic, molecular and cellular scales.

## Main

Protein synthesis, translation, is an essential process for every living cell and is, thus, one of the major targets for antimicrobial drugs^1,2^. Chloramphenicol (Cm) was the first broad-spectrum antibiotic to be clinically used. It inhibits translation through binding to the peptidyl transferase center (PTC) in the large subunit (LSU) of the bacterial ribosome^1–3^. Structural studies suggest that Cm blocks new peptide bond formation by sterically hindering the positioning of the aminoacyl moiety of the A-site transfer RNA (tRNA) in the PTC^4–6^. Yet, in contrast to the longstanding notion that Cm is a general inhibitor of translation, recent evidence from ribosome profiling and toeprinting indicates that Cm and similar PTC-binding antibiotics such as linezolid block translation in a manner that depends on the amino acid sequence of the nascent peptide in the PTC^7^. Cm preferentially inhibits peptidyl transfer when the penultimate residue of the nascent peptide (position −1, where position 0 is defined as that attached to the P-site tRNA) is Ala or, to a lower extent, Ser and Thr. The presence of Asp at position 0 or Lys at position −3 potentiates Cm inhibition. Conversely, Cm shows almost no inhibition when Gly is at position 0 or in the incoming aminoacyl-tRNA (aa-tRNA; position +1) in the A-site^7^. These findings were confirmed by in vitro single-molecule fluorescence experiments, showing that Cm does not inhibit translation until the arresting sequence motifs are synthesized and that inhibition is circumnavigated when the incoming aa-tRNA carries Gly^8^. Single-molecule tracking of translation kinetics in *Escherichia coli* cells treated with Cm also indicated slow but ongoing translation^9^. Thus, despite Cm being one of the most extensively studied antibiotics, how the new evidence on its context-dependent mechanism of action is manifested at a structural level remained an open question.

To address this gap, recent in vitro structures of *Thermus thermophilus* ribosomes in the presence of nonhydrolyzable tripeptidyl-tRNA analogs as P-site ligands revealed favorable context-dependent interactions between Cm and amino acid residues of the nascent peptide, especially Ala at position −1, which are required to stabilize Cm in the PTC^10,11^. A similar sequence-dependent inhibition mechanism is also postulated for oxazolidinone antibiotics that bind at a PTC site overlapping with Cm (ref.^12^). However, because fragments mimicking aa-tRNA outcompete Cm from its canonical binding site^10^ and only A-site-bound Gly-tRNA is compatible with simultaneous Cm binding^11^, the structure of a Cm-stalled ribosome in complex with full peptidyl-tRNA in the P-site and aa-tRNA in the A-site remains elusive. Furthermore, in vitro experiments with defined short peptide mimics cannot recapitulate the inhibition of translation of the plethora of mRNAs inside living cells.

In this study, we used cryo-electron tomography (cryo-ET) to image intact *Mycoplasma pneumoniae* cells treated with Cm and obtained in-cell ribosome maps at better than 3.0 Å local resolution through subtomogram analysis. This enabled us to analyze the interaction of Cm with translating ribosomes in detail and decipher the impact of Cm on the translation processes across scales within the native cellular context.

## Results

### High-resolution features of ribosomes in bacterial cells

**Fig. 1:**
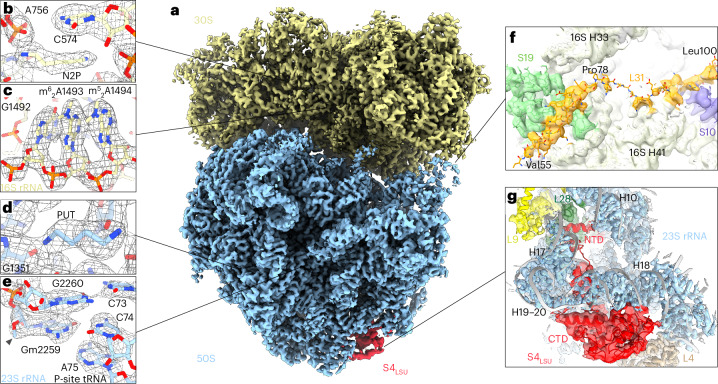
Ribosome maps and models in Cm-treated *M.* *pneumoniae* cells. **a**, Composite of the focused refined 50S and 30S maps of the *M.* *pneumoniae* 70S ribosome. **b**–**e**, Representative high-resolution features of the polyamines cadaverine (**b**; N2P), putrescine (**d**; PUT) and several RNA-base modifications of 16S rRNA (**c**; m^6^_2_A:*N*^6^,*N*^6^-dimethyladenosine) and 23S rRNA (**e**; Gm: 2′-*O*-methylguanosine, arrowhead). **f**, The CTD of the ribosomal protein L31 was modeled from Val55 to Leu100. The interacting rRNA and ribosomal proteins are indicated. **g**, A second copy of ribosomal protein S4 was resolved and identified on the LSU in 33% of 70S ribosomes after focused classification. The map and model around S4_LSU_ are shown.

**Extended Data Fig. 1:**
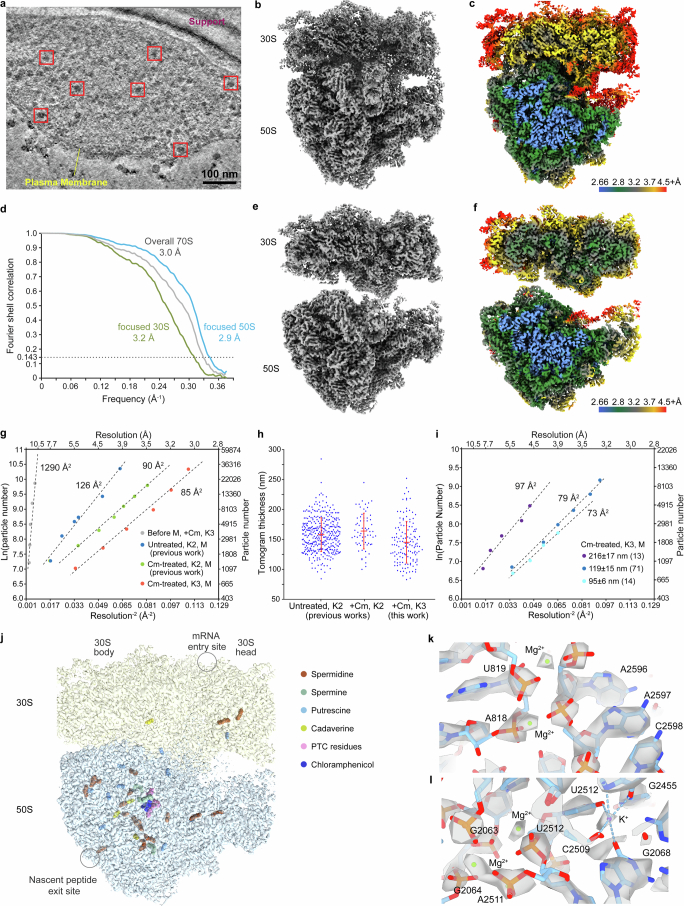
Ribosome sub-tomogram analysis of Cm-treated *M. pneumoniae* cells. **a**, Slice of a denoised representative cellular cryo-tomogram of *M. pneumoniae*. Several ribosomes (red boxes), the plasma membrane, and edge of the grid support film are annotated. **b-c**, The 70S ribosome consensus map (**b**), and colored by local resolution (**c**, scale shown on bottom right). **d**, Fourier Shell Correlation (FSC) curves for the 70S ribosome consensus map, the small (30S) and large (50S) subunits after focused refinements on the 70S ribosome consensus map. **e**-**f**, Maps of the small and large subunits after focused refinements on the 70S ribosome consensus map (**e**), and colored by local resolution (**f**). **g**, B factor plots for the dataset processed in the current work (Before M, +Cm, K3; Cm-treated, K3, M) in comparison to our previously published untreated and Cm-treated *M. pneumoniae* ribosome data (collected with a K2 camera) after M refinement^15^. **h**, Comparison of cell thickness distribution for the three datasets described in g. Each dot represents a cell. Red lines indicate mean and standard deviation. Cell thickness for the dataset described in this work is 145 ± 35 nm (n = 137 cells). The thicknesses of 356 and 65 cells are plotted for the previously published data of untreated and Cm-treated datasets^15^. **i**, B factor plots for selected tomogram subsets of different cell thickness form the current dataset. The cell number (in brackets), mean thickness and standard deviation values are shown. Analysis of a subset of cells with thickness around 100 nm achieved a B factor lower than 80 Å^2^. **j**, Overview of polyamines resolved and modeled in the ribosome structure. **k**, Two Mg^2+^ ions coordinated by 23S rRNA nucleotides^30^. **l**, Region near the PTC showing rRNA nucleotides and ions. Five coordination bonds for the K^+^ ion can be mapped^37^. Source data

Cryo-ET data of *M.* *pneumoniae* cells treated with Cm for 15 min were acquired at a pixel size of 1.33 Å, with each tomogram capturing the majority of one cell (Methods). Subjecting 30,774 ribosome-containing subtomograms from 137 cells to structural analysis resulted in a 70S ribosome map at 3.0 Å global resolution, with an overall *B* factor^13^ of 85 Å^2^ (Extended Data Fig. 1a–d,g–i). Focused refinements on the LSU (50S) and small subunit (SSU; 30S) of the ribosome provided maps at 2.9 Å (with the core resolved to the Nyquist limit) and 3.2 Å, respectively (Fig. 1a and Extended Data Fig. 1d–f). The enhanced resolution of the maps, compared to our previous work^14,15^, allowed us to improve the atomic model of the *M.* *pneumoniae* ribosome (Fig. 1, Table 1 and Methods). For instance, we modeled a number of naturally bound polyamine molecules, namely, cadaverine, putrescine, spermine and spermidine, as well as magnesium and potassium ions (Fig. 1b,d, Extended Data Fig. 1j–l and Extended Data Table 1). Polyamines were shown to bind to ribosomes in in vitro studies, and are important for stabilization of the ribosomal RNA (rRNA) structure and for translation regulation^16^. Our structural models demonstrate the natural composition of polyamines associated with ribosomes inside *M.* *pneumoniae* cells. While *M.* *pneumoniae* lost essential enzymes to synthesize polyamines, it preserves membrane transporters responsible for polyamines uptake from the environment or host^17,18^. We also identified and modeled several rRNA base modifications (Fig. 1c,e and Extended Data Table 1), of which six aligned with the modifications identified in cryo-electron microscopy (cryo-EM) and X-ray maps of isolated *E. coli* or *T. thermophilus* ribosomes^19–22^. We found one base to be differently modified (16S rRNA m^4^C1377 versus m^4^Cm1402 in *E.* *coli* and *T.* *thermophilus*) and one to be unique to *M.* *pneumoniae* (23S rRNA m^1^G783 versus unmodified G750 in *E.* *coli*). In addition, the C-terminal domains (CTDs) of ribosomal proteins S6 (residues 131–184) and L31 (residues 47–100) were better resolved in the new maps and could be correctly modeled (Fig. 1f). The proteins L31 and S19 form the intersubunit bridge B1c in bacterial ribosomes^23^. Interestingly, the 20 most C-terminal residues of L31 (an extension in *M.* *pneumoniae* compared to other bacteria^15^) reach even further to contact protein S10, thereby strengthening the intersubunit bridge.

**Table 1:** Cryo-ET data collection and structure refinement statistics of high-resolution ribosome averages in Cm-treated cells

	70S ribosome average (EMD-17132), (PDB 8P7X)	30S SSU (EMD-17133), (PDB 8P6P)	50S LSU (EMD-17134), (PDB 8P8B)	70S with S4_LSU_ (EMD-17135), (PDB 8P7Y)
**Data collection and processing**				
Magnification	64,000			
Voltage (kV)	300			
Electron exposure (e^−^ per Å^2^)	137			
Electron exposure per tilt image (e^−^ per Å^2^)	3.34			
Energy filter slit width (eV)	20			
Tilt range and scheme	−60°:3°:60°; dose symmetric			
Defocus range (μm)	−1.0 to −3.25			
Pixel size (Å)	1.329			
Symmetry imposed	*C1*			
Initial subtomograms (no.)	30,774	30,774	30,774	30,774
Final subtomograms (no.)	30,774	30,774	30,774	10,151
Map resolution (Å)				
FSC threshold = 0.143	3.0	3.2	2.9	3.7
Map resolution range (Å)	2.8 to 6	3 to 5	2.8 to 4	3.2 to 6
Map sharpening *B* factor (Å^2^)	−1 to +15	−1 to +15	−1 to +15	−1 to +15
**Model refinement**				
Initial model used (PDB code)	7OOC, 7OOD	7OOC	7OOD	7OOC, 7OOD
Model resolution (Å)				
FSC threshold = 0.143	3.02	3.28	3.08	3.62
Model resolution (Å)				
FSC threshold = 0.5	3.28	3.60	3.16	4.04
Model versus map correlation coefficient	0.73	0.74	0.80	0.73
Model composition				
Nonhydrogen atoms	151,587	55,468	97,171	153,258
Protein residues	6,182	2,597	3,578	6,386
RNA residues	4,755	1,612	3,194	4,755
Ligands and water	367	112	266	369
*B* factors (Å^2^) (mean)				
Protein	70.84	82.90	62.04	71.55
RNA	73.77	88.29	67.03	73.77
Ligands	31.27	29.99	31.44	31.27
Water	-	30.00	-	-
R.m.s.d.				
Bond lengths (Å)	0.003	0.003	0.004	0.005
Bond angles (°)	0.580	0.555	0.592	0.750
**Validation**				
MolProbity score	1.93	2.03	2.20	2.10
Clashscore	8.98	10.60	7.63	13.04
Poor rotamers (%)	0.83	0.58	3.75	1.00
Ramachandran plot				
Favored (%)	92.84	91.85	95.02	92.23
Allowed (%)	6.71	7.72	4.89	7.14
Disallowed (%)	0.44	0.43	0.09	0.64
**Validation (RNA)**				
Good sugar pucker (%)	99.20	99.50	99.53	99.26
Good backbone (%)	81.42	84.40	81.70	81.49

**Extended Data Fig. 2:**
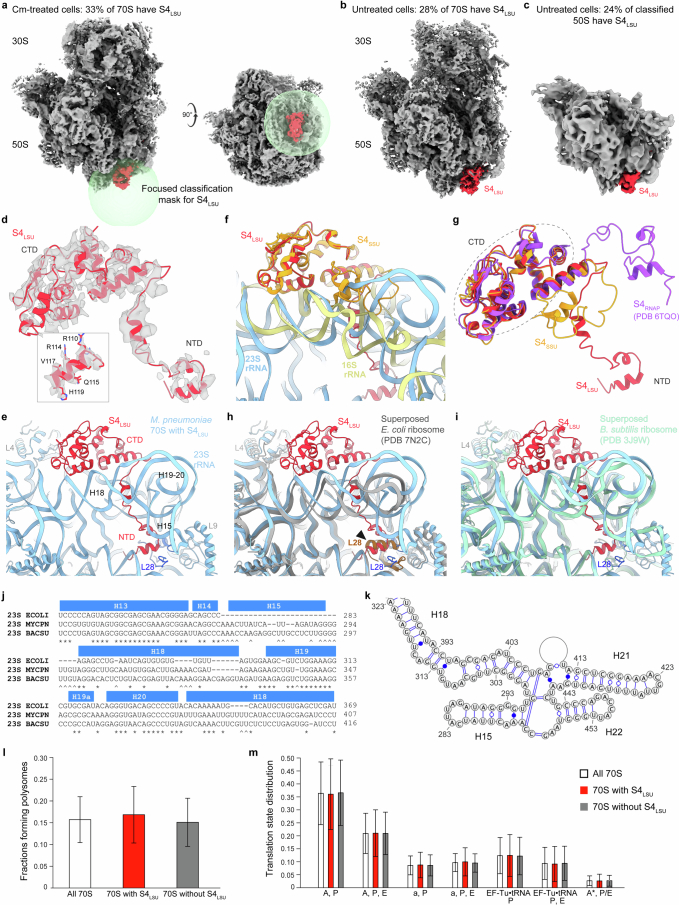
*M. pneumoniae* ribosomes with additional S4_LSU._ **a**, Mask (green sphere) used for focused classification of ribosomes with S4_LSU_ (red) in Cm-treated cells. **b-c**, S4_LSU_ detected in 70S (**b**) and free 50S (**c**) in native untreated cells. **d**, S4_LSU_ model fitted into the corresponding density in the Cm-treated ribosome map. Inset: Map and model for a short helix of S4_LSU_ showing the quality of the map allowing robust model building. **e**, Region of the ribosome structure with S4_LSU_ which contacts 23S rRNA helices 15, 18–20 and 52, as well as protein L28. **f**, Comparison of the canonical S4 (S4_SSU_) binding site on the small ribosomal subunit (yellow) and the S4_LSU_ binding site (blue, aligned on S4). **g**, Comparison of different conformations of S4 in the RNA polymerase anti-termination complex (purple), the small (yellow) and large ribosomal subunits (red). Models aligned on S4 CTD show the different conformations of the NTDs. **h**, S4_LSU_ N-terminal helix would clash (black arrowhead) with L28’s C-terminal helix of a superposed *E. coli* ribosome which has 13 additional residues in the C-terminus compared to *M. pneumoniae*. **i**, *B. subtilis* L28 does not have a C-terminal extension and could accommodate the superposed S4_LSU_. It also harbors 23S rRNA helix 15 of a similar length to *M. pneumoniae*. **j**, Sequence alignment of 23S rRNA helices 13–20 of *E. coli, B. subtilis* and *M. pneumoniae*, showing the insertion of helix 15 in *M. pneumoniae* and *B. subtilis* (see also panels **e** and **h**). Carets mark sequence conservation in two and asterisks in all three. **k**, Secondary structure plot of 23S rRNA helices 14–22 in *M. pneumoniae*. **l-m**, Polysome engagement (**l**) and distribution of translation elongation states (**m**) of the total 70S ribosomes in Cm-treated cells, and those with or without S4_LSU_. Bars represent mean fractions and whiskers represent standard deviations across 137 cells. Source data

Surprisingly, in approximately one third of all 70S ribosomes, we identified a second copy of the small ribosomal protein S4 bound to the LSU (hereafter called S4_LSU_; Fig. 1g, Extended Data Fig. 2a–e and Methods). The conformation of this additional S4_LSU_ differs from that of the canonical S4 in the SSU or S4 in the RNA polymerase antitermination complex^24^ (Extended Data Fig. 2f,g). The S4_LSU_ binding site is located on helices 12, 13 and 18 of 23S rRNA, a site that has not been previously reported as a factor association site in bacterial ribosomes and is far away (about 200 Å) from S4’s canonical binding site near the mRNA entry channel. The flexible N-terminal domain (NTD) of S4_LSU_ (residues 1–30) protrudes into a cavity located below 23S rRNA helix 18 and contacts protein L28 (Fig. 1g and Extended Data Fig. 2e). The outer perimeter of the cavity is formed by 23S rRNA helix 15, which is similar to that in *Bacillus subtilis* in length and conformation but is missing in *E.* *coli* (Extended Data Fig. 2h–k). Ribosomes with the S4_LSU_ showed no obvious difference in their distribution across translational states or polysome association compared to the overall 70S population (Extended Data Fig. 2l,m). The discovery of S4_LSU_ made here prompted us to examine our previously published data on untreated *M.* *pneumoniae*^15^ for the presence of an extra copy of S4. In fact, we found S4_LSU_ in both 70S and free 50S after focused classification of the untreated *M.* *pneumoniae* ribosomes, with similar occurrence frequency and overall structure (Extended Data Fig. 2b,c). S4_LSU_, thus, appears to naturally occur in *M.* *pneumoniae* ribosomes, while its function remains elusive (Discussion).

### Cm interacts with the native nascent peptide in the PTC

**Fig. 2:**
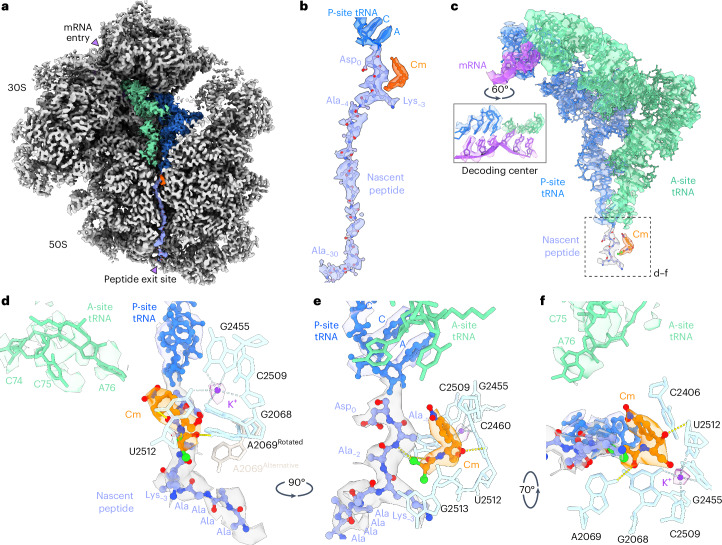
The Cm-binding site in the PTC is shaped by 23S rRNA, ions and the nascent peptide. **a**, A composite in-cell ribosome map resolves mRNA, tRNAs and the native nascent peptide. Differently sharpened and blurred maps were combined to visualize the nascent peptide density (blue gray) from the PTC to the peptide exit site. **b**, Zoomed-in view for the nascent peptide density from the PTC to the exit site. **c**, Atomic model for mRNA (purple), A-site and P-site tRNAs (green and blue, respectively), nascent peptide (blue gray) and Cm (orange). The solid rectangle denotes the codon–anticodon pairing in the decoding center. The dashed rectangle denotes the Cm-binding site, described at higher detail in **d**–**f**. **d**, The Cm-binding pocket in the A-site of the PTC is formed by 23S rRNA nucleotides (cyan; only a few bases are displayed) and the nascent peptide (blue gray). For clarity, only positions 0 to −7 of the nascent peptide are shown. The first four residues (Asp-Ala-Ala-Lys) were modeled in accordance to a ribosome profiling study^7^. Density (purple) near the Cm-binding site was resolved and modeled as a K^+^ ion on the basis of previous studies^30,37^. The CCA tail of aa-tRNA in the A-site (green) is shown with the corresponding density in **d** and **f**. **e**, Side view of the Cm-binding pocket. **f**, Top view of the binding pocket.

**Extended Data Fig. 3:**
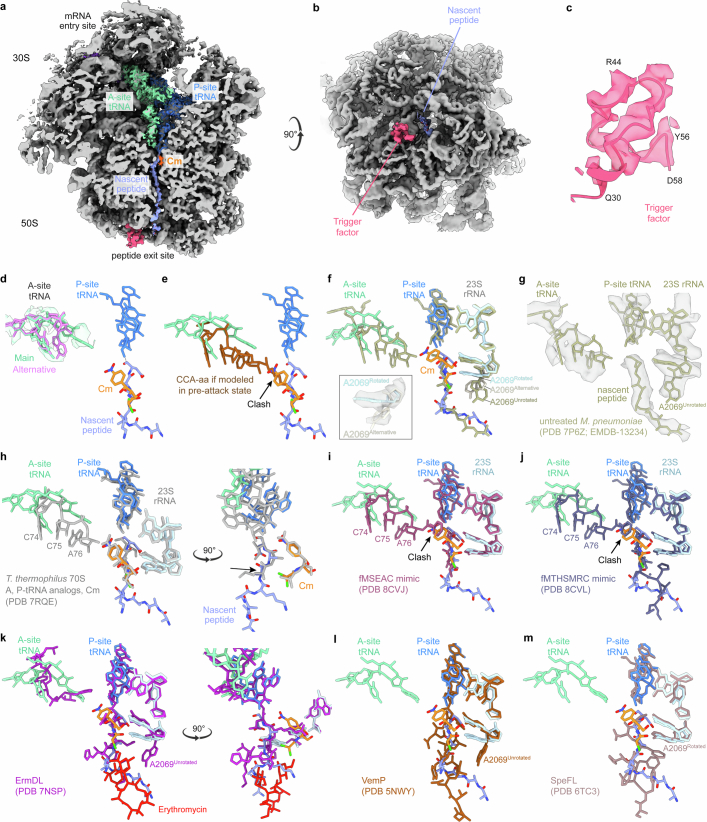
Comparison of the PTC in the presence of Cm across different structures. **a**, Composite map of the Cm-bound ribosome. Positive B factor was used to blur the map (Methods). **b**, View from the peptide exit site showing the modeled trigger factor (pink). **c**, Map and model of the N-terminal ribosome binding domain (residues 29–58) of trigger factor. **d**, Two possible modeling solutions for the CCA-tail (main, green; alternative, magenta) of the aminoacyl-tRNA in the A-site. **e**, Clash (arrow) of the incoming amino acid of A-site tRNA (brown) with Cm (orange) if modeled in the pre-attack state. **f**, Superposition of 70S model from untreated *M. pneumoniae* cells^15^ (yellow-gray) with the Cm-treated 70S model determined in this study. Inset: A2069 in Cm-treated ribosome has an alternative conformation (light gray), similar to the unrotated conformation in the untreated structure. **g**, 70S map and model from untreated *M. pneumoniae*. **h**, Comparison of the Cm-treated 70S model determined in this study with *in vitro* Cm-treated *T. thermophilus* ribosome (grey) with deacylated tRNA analog in A-site and non-hydrolyzable peptidyl-tRNA analog that mimics the nascent peptide up to position −2 (black arrow on right). **i-j**, Comparison of the Cm-treated 70S model determined in this study with *in vitro T. thermophilus* 70S structures with non-hydrolyzable aminoacyl-tRNA^Phe^ and peptidyl-tRNA analogs that mimic the peptide sequences fMSEAC (**i**, purple) and fMTHSMRC (**j**, grey blue). **k-m**, Comparison of the Cm-treated 70S model determined in this study with *in vitro* structures of stalled ribosome-nascent peptide complexes: ErmDL with erythromycin (**k**, dark purple), VemP (**l**, sienna); SpeFL (**m**, tan).

The in-cell consensus map resolved to the data limit of 2.7 Å at the 50S core enabled us to investigate Cm’s binding and coordination in atomic detail in the context of stalled native translation complexes. Cm was clearly resolved in the A-site of the PTC (Fig. 2a–c), its canonical binding site, consistent with previous in vitro structures^4–6,10,11^. Ribosomal cofactors, including mRNA, aa-tRNA in the A-site and the natural peptidyl-tRNA in the P-site (in contrast to the synthetic peptide analogs required to generate in vitro structures^10,11^) could be modeled with high confidence (Fig. 2b,c, Extended Data Fig. 3a–c and Methods). We found the body of aa-tRNA to be fully accommodated, except for its CCA tail carrying the incoming amino acid, which was positioned further away from the A-site cleft because of Cm’s steric hindrance (Fig. 2d–f; detailed comparison between different structures is provided in Extended Data Fig. 3d–f). The blurred local density indicated that the CCA tail is not stably positioned and may adopt different conformations (Extended Data Fig. 3d). Connecting to the CCA tail of the P-site tRNA, the nascent peptide could be traced from the PTC to the peptide exit site, with side-chain densities resolved for amino acid residues from positions 0 to −3 (Fig. 2b,d–f). Hence, amino acid residues at positions 0, −1 and −3 were built as Asp, Ala and Lys, in line with the sequence reported to be overrepresented in Cm-stalled ribosomes^7^. The remaining nascent peptide after position −4 could be traced only for the backbone and was modeled as poly(Ala) (labeled as poly(UNK) in the model).

In the PTC, 23S rRNA nucleotides (A2459, C2460, G2068, A2069 and U2512) form the binding pocket for Cm (Fig. 2d–f). The base of C2460 (C2452 in *T.* *thermophilus* and *E.* *coli*) and Cm’s nitrobenzene ring interacts through *π* stacking (Fig. 2e). The base of A2069 (A2062 in *T.* *thermophilus* and *E.* *coli*) rotates by more than 120° upon Cm binding when compared to the untreated *M.* *pneumoniae* ribosome structure^15^ (Extended Data Fig. 3f,g), consistent with previous observations^4–6^. In the rotated conformation, A2069 forms hydrogen bonds with Cm’s carboxyl group and the main chain N atom of the residue at position −2 (Fig. 2d,f). We also observed additional density corresponding to other possible rotamers of A2069 (Extended Data Fig. 3f–m), reflecting its dynamic nature.

In addition to 23S rRNA, we found that the elongating nascent peptide directly interacts with Cm. The side chain of the residue at position −1 forms a CH–*π* interaction with the nitrophenyl ring of Cm and the residue’s main chain N atom also forms a hydrogen bond with the CL1 atom of Cm (Fig. 2e). This model supports a central role of the residue at position −1 in the interaction with Cm, consistent with previous in vitro structures^10^. Moreover, we found that the residue at position −3 is involved in shaping the Cm-binding pocket in the native translation complex (Fig. 2e), restricting the pocket at the side facing the nascent peptide tunnel. We modeled the nascent chain residue at position −3 as Lys, consistent with a previous functional study^7^ that revealed a more pronounced translation inhibition by Cm upon accumulation of Ala at position −1 and Lys at position −3. The lysyl side chain is located at a distance of about 3.5 Å from Cm’s dichloroacetyl group and can be stabilized by aliphatic–aromatic stacking on 23S rRNA nucleotide G2513. From position −4 onward, the nascent peptide is kinked by nearly 90°, which places it far away from the PTC and the Cm-binding site (Fig. 2d,e and Extended Data Fig. 3f,g).

In *T.* *thermophilus*, Cm was postulated to interact through its (methylene) C4 hydroxyl group with a potassium ion in the PTC (2.7 Å distance between Cm’s O4 and K^+^; Protein Data Bank (PDB) 4V7W (ref. ^4^)). We were able to place a corresponding K^+^ ion, coordinated by 23S rRNA nucleotides U2512, G2068, G2455 and C2509, 4.1 Å away from Cm’s methylene hydroxyl (O4::K^+^ distance; Fig. 2d–f). This distance does not allow for direct ion coordination but implies an indirect interaction through a water molecule, in agreement with other Cm-bound *T.* *thermophilus* ribosome structures (4.1 Å for PDB 6ND5 (ref. ^6^) and 4.25 Å for PDB 7U2J (ref. ^11^)).

In summary, our in-cell structural model provides detailed information about how Cm binds and reshapes the PTC, and elucidates its interactions with the natural nascent peptide that give rise to the recently postulated sequence-dependent inhibition mechanisms of PTC-binding antibiotics.

### Cm enriches multiple translation elongation intermediates

**Fig. 3:**
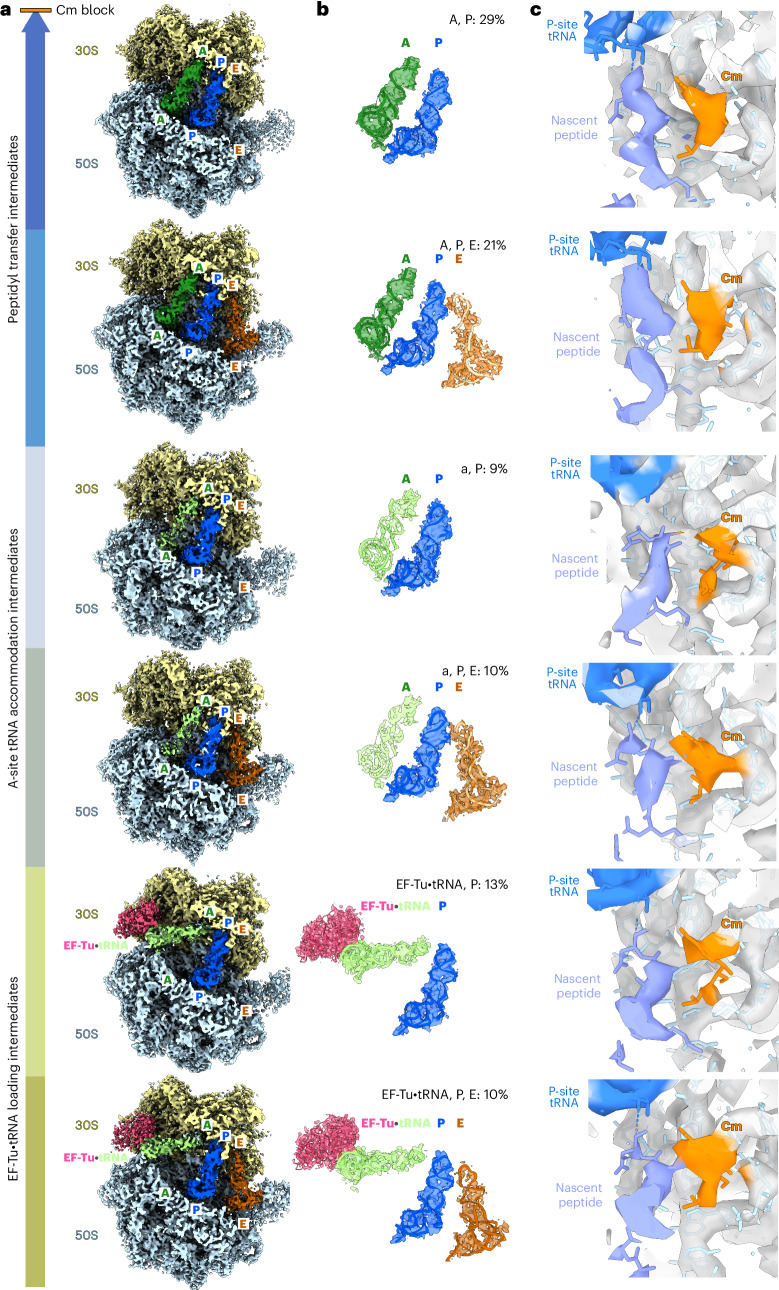
Cm enriches sequential translation elongation intermediates before peptidyl transfer. **a**, Density maps of the seven translation elongation intermediates (six of these are shown) are characterized by differences in tRNA binding (light green, A/T-site; dark green, A-site; blue, P-site; brown, E-site) and the elongation factor EF-Tu (pink). The seventh rotated pretranslocational class ‘A*, P/E’ is not shown here (detailed in Extended Data Fig. 4i). **b**, Density maps and models of the tRNAs and elongation factor identified in the translation elongation intermediates. A unique class name, for example ‘A, P, E’, is given to each class on the basis of tRNAs and EF-Tu occupancy. The percentage of each class was calculated based on particle numbers. The lower case ‘a’ refers to the flexible aa-tRNA near the A-site, which is only partially resolved (detailed in Extended Data Fig. 4g). **c**, Cm (orange for density and fitted molecule) was resolved in all six classes, with the corresponding density observed in the canonical binding site in the PTC after fitting the model. Source data

**Extended Data Fig. 4:**
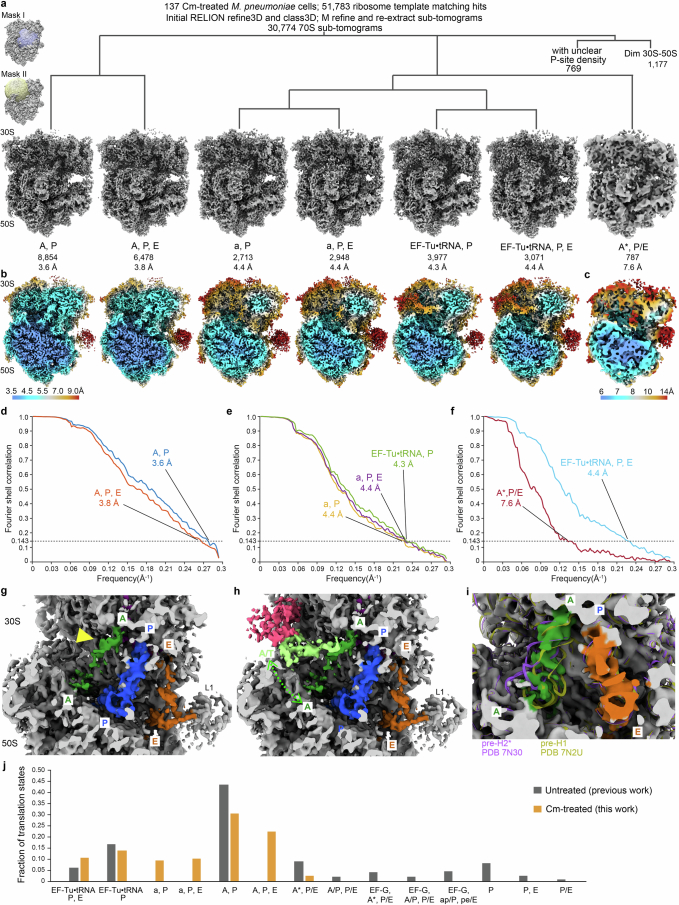
Classification of ribosomes translation elongation states in Cm-treated *M. pneumoniae* cells. **a**, The cryo-ET data processing and sub-tomogram classification workflow. At least three tiers of classifications were performed, including global 70S classification, focused classification on A-P-E-tRNA association sites (mask I), and on elongation factor and A/T-tRNA binding region (mask II). For each class, the average map, a class name, particle numbers and the global resolution (FSC = 0.143) are provided. Two classes generated at the first tier of global 70S classification (769 and 1,177 particles) resulted in low-resolution ribosome maps with unexplainable densities and are not shown. These are denoted as ‘unclear’ in Extended Data Fig. 5. **b**, Local resolution maps (color coded according to the bar on bottom left) for the six major classes. **c**, Local resolution map (color coded according to the bar on bottom right) for the minor class. **d-f**, FSC curves for all classes based on RELION postprocessing. **g-h**, In the ‘a, P’ and ‘a, P, E’ classes, the A-site tRNA’s anticodon end bound to the 30S decoding center is well-resolved (yellow arrowhead), but the body shows blurred density. They possibly represent 70S intermediates with the incoming tRNA sampling between the A/T- and the classic A-site (as illustrated in **h**). Maps were low-pass filtered to 6 Å for comparison. **i**, The ‘A*, P/E’ minor class represents intermediates between the pre-translocational hybrid H1 and H2* states^25^. Models were fitted in the map for comparison. **j**, Comparison of the distribution of the classified translation elongations states of 70S ribosomes in untreated^15^ and Cm-treated cells. Source data

To assess the impact of Cm on the translation process, we performed structural classification of the 70S ribosomes in the Cm-treated cells. We identified seven highly populated translation elongation intermediates, among which six were determined at better than 4.5 Å resolution, allowing unambiguous assessment of the presence of the Cm molecule in each of the states (Fig. 3, Extended Data Fig. 4a–f and Table 2). We found about 50% of all 70S ribosomes to be stalled in the classical pretranslocational states ‘A, P’ and ‘A, P, E’ (named according to tRNA occupancy). As in the consensus map (Fig. 2), the A-site tRNA was fully accommodated except for its CCA tail, because of a steric clash of Cm with the incoming aa-tRNA. Accordingly, peptidyl transfer cannot occur^1,2^. In addition, 19% of 70S showed a weak tRNA density near the A-site (classes ‘a, P’ and ‘a, P, E’), in which only the tRNA anticodon loop bound to mRNA in the 30S decoding center showed clear density, whereas the tRNA main body density was blurred (Fig. 3b and Extended Data Fig. 4g). The blurred local density suggests that the incoming aa-tRNA possibly swings between the A/T-site and A-site (Extended Data Fig. 4h). Two additional decoding intermediates with bound EF-Tu•tRNA (classes ‘EF-Tu•tRNA, P’ and ‘EF-Tu•tRNA, P, E’) accounted for 23% of the ribosomes, similar to the fraction classified in untreated cells^15^ (Extended Data Fig. 4j). Blurred local density for EF-Tu suggests that the respective subpopulation is a mixture of decoding intermediates, which could not be further classified. The above-described six intermediates can be aligned along the translation elongation trajectory before new peptide bond formation (Fig. 3a). Remarkably, they all showed Cm density in their PTCs (Fig. 3c).

**Table 2:** Cryo-ET data processing and structure refinement statistics of 70S ribosome and disome classes in Cm-treated cells

	70S class ‘A, P’	70S class ‘A, P, E’	70S class ‘a, P’	70S class ‘a, P, E’	70S class ‘EF-Tu•tRNA, P’	70S class ‘EF-Tu•tRNA, P, E’	70S class ‘A*, P/E’	Disome class I	Disome class II	Disome class III	
										PDB 8P8V (leading ribosome)	PDB 8P8W (following ribosome)
	EMD-17136	EMD-17137	EMD-17138	EMD-17139	EMD-17140	EMD-17141	EMD-17142	EMD-17143	EMD-17144	EMD-17145	
**Data collection and processing**^a^											
Final subtomogram (no.)	8,854	6,478	2,713	2,948	3,977	3,071	787	654	387	963	
Map resolution (Å)											
FSC threshold = 0.143	3.6	3.8	4.4	4.4	4.4	4.4	7.6	10	15	8.7	
Map resolution range (Å)	3.2 to 4.2	3.4 to 4.5	4 to 6	4 to 6	4 to 8	4 to 8	6 to 10	6 to 25	8 to 25	5 to 15	
*B* factor (Å^2^)	−7	−5	−8	−8	−1	−5	−22	−100	−150	−50	
**Model refinement**											
Initial model used (PDB code)										7OOC, 7OOD	
Model resolution (Å)											
FSC threshold = 0.143										8.52	8.63
Model resolution (Å)											
FSC threshold = 0.5										8.92	11.10
Model versus map correlation coefficient										0.76	0.60
Model composition											
Nonhydrogen atoms										153,639	151,556
Protein residues										6,436	6,394
RNA residues										4,773	4,688
Ligands										334	316
*B* factors (Å^2^) (mean)											
Protein										71.46	71.48
RNA										76.15	77.10
Ligand										35.81	38.68
R.m.s.d.											
Bond lengths (Å)										0.004	0.003
Bond angles (°)										0.721	0.638
**Validation**											
MolProbity score										2.20	2.17
Clashscore										16.58	14.33
Poor rotamers (%)										0.07	0.00
Ramachandran plot											
Favored (%)										92.18	91.42
Allowed (%)										7.02	7.64
Disallowed (%)										0.81	0.94
**Validation (RNA)**											
Good sugar pucker (%)										99.22	99.25
Good backbone (%)										81.14	81.24

^a^Data collection parameters are specified in Table 1.

In addition, we identified a rotated pretranslocational intermediate (‘A*, P/E’) with a marginally translocated A-site tRNA and a hybrid P/E-site tRNA, which accounted for 2.6% of the 70S ribosomes and was refined to 7.6 Å resolution (Extended Data Fig. 4i). The fragmented density for tRNA in the A-site indicates that this subpopulation is a mixture of rotated-1 and rotated-2 pretranslocational states (also called hybrid states H2* and H1, respectively)^25^. While we could not determine whether Cm is bound in the PTC because of the low resolution of this map, formation of the rotated pretranslocational states with hybrid P/E-site tRNA clearly requires successful peptidyl transfer. This intermediate could, therefore, represent a fraction of ribosomes where Cm did not inhibit peptide bond formation because of the presence of a contextually disfavored residue such as Gly at position 0 of the nascent peptide^7^.

In summary, we found that, inside living cells, Cm binding to ribosomes enriches for a number of sequential translation elongation intermediates before new peptide bond formation, in contrast to the expected accumulation of a single ‘A, P’ state.

### Cm leads to polysome reorganization and collisions

**Fig. 4:**
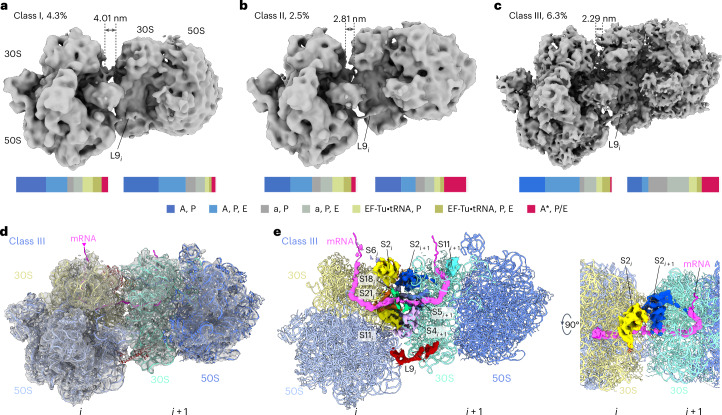
Cm induces ribosome collisions within polysomes. **a**–**c**, Three different ribosome pair (disome) arrangements found in *M.* *pneumoniae* cells treated with Cm: disome classes I (**a**), II (**b**) and III (**c**), each shown with its percentage among all 70S ribosomes and mRNA exit-to-entry distance (dashed lines). Lower bars, the distributions of translation elongation states for the leading (left) and the following (right) ribosomes of each disome class are calculated based on the ribosome state classification results (Extended Data Fig. 4a). **d**,**e**, Map (**d**) and atomic model (**e**) of disome class III. The major ribosomal proteins at the interface are S2, S6, S18, S21, S11 and L9 of the leading ribosome (*i*) and S2, S5 and S4 of the following ribosome (*i* + 1). Ribosomal protein S1 is not found in *M.* *pneumoniae*^17^. The mRNA (pink) path can be traced threading between ribosomal proteins S21 and S18 of the leading ribosome and S4 and S5 of the following one. Source data

**Extended Data Fig. 5:**
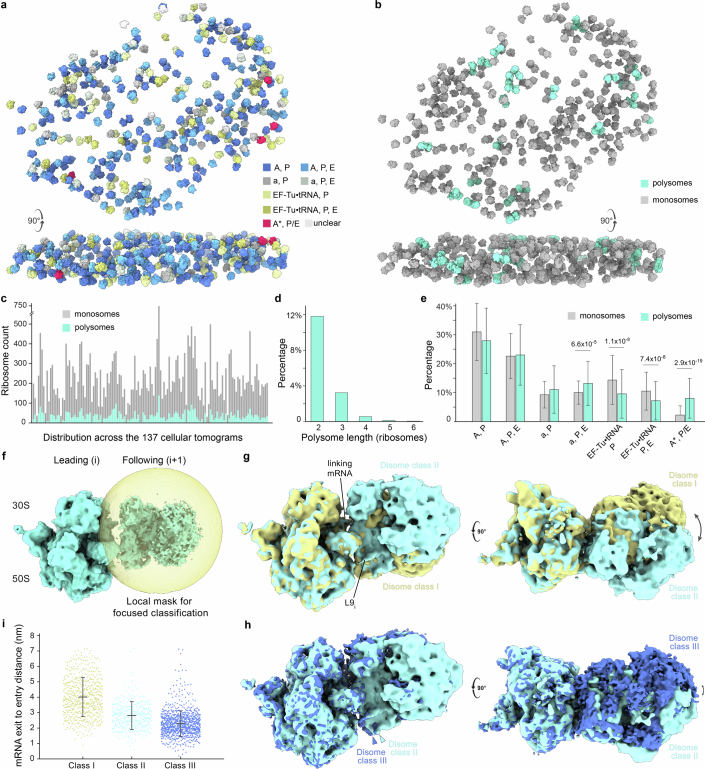
Spatial and structural analysis of polysomes in Cm-treated *M. pneumoniae* cells. **a**, An example of ribosomes of different functional states classified as described in Extended Data Fig. 4a mapped into the 3D cellular volume. **b**, The same cell as in panel **a** colored according to the polysome detection results (light green: polysomes, gray: monosomes). **c**, Counts of monosomes and polysomes across the 137 cellular tomograms. **d**, Distribution of polysomes length (number of ribosomes). **e**, Distribution of translation elongation states in monosomes and polysomes. Bars represent mean percentages and whiskers represent standard deviations across the 137 cells. False discovery rate-adjusted *p* value (two-sided Wilcoxon rank sum test) is provided. **f**, The local mask (yellow sphere) used to classify neighboring ribosome pairs (disomes) within the 4,838 spatially annotated polysomes. Aligned on the leading ribosome. **g**, Comparison of disome classes I and II, after alignment on the leading ribosome (left). The major difference comes from the relative rotation of the following ribosome. **h**, Comparison of disome classes II and III. The minor difference comes from the displacement and relative rotation of the following ribosome. **i**, Distribution of the mRNA exit_i_-to-entry_i+1_ distances for the three disome classes classified in RELION. The mean (line) and standard deviation (whiskers) of distances for the three disome classes are 4.01 ± 1.27 nm (n = 654 disomes), 2.81 ± 0.91 nm (n = 387), 2.29 ± 0.83 nm (n = 963). Source data

To probe how Cm influences translation at the cellular level, we next performed spatial analysis of the ribosome classes mapped back into the three-dimensional (3D) cellular volumes and, in particular, assessed their arrangement in polysomes (Methods). In comparison to untreated cells where 26.2% of 70S ribosomes were found to associate in closely assembled polysomes^15^, only 15.7% of ribosomes were annotated as polysomes in the Cm-treated cells (Extended Data Fig. 5a–e and Extended Data Table 2). In the Cm-treated cells, 91.3% of the ribosome pairs (disomes) within the polysomes exhibited the compact ‘top–top’ (t–t) configuration^15,26^ (Fig. 4). Disome subtomograms were extracted and subjected to classification, resulting in the determination of three distinct arrangements (classes I, II and III; Fig. 4a–c, Extended Data Fig. 5f–h and Table 2). The three classes differed in the relative rotation and positioning of the following ribosome with respect to the leading one, which can be reflected by the distance from the leader’s mRNA exit to the follower’s mRNA entry site (Extended Data Fig. 5i).

**Extended Data Fig. 6:**
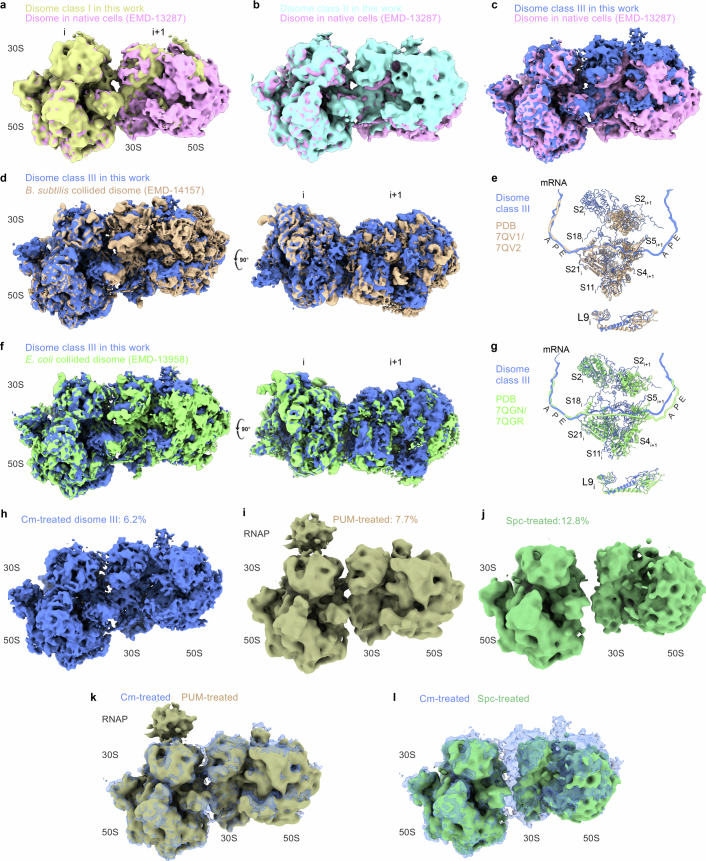
Comparison of disome arrangements across different conditions. **a-c**, The Cm-treated disome class I is structurally similar to disomes in native untreated *M. pneumoniae* cells^15^ (**a**), while disome classes II (**b**) and III (**c**) are more compact. The best resolved disome class in untreated cells (pink, EMD-13287) is shown. All maps are aligned on the leading ribosome (left) for comparison. **d-g**, Cm-treated disome class III (blue) resembles the *in vitro* reconstituted collided disomes of *B. subtilis*^27^(**d, e**) and *E. coli*^28^(**f**, **g**). Aligned on the leading ribosome for comparison. The positioning of mRNA and interface ribosomal proteins largely overlap (**e, g**). There is a slight rotational difference of the L9 protein. **h-l**, Comparison of the Cm-treated disome class III to disome structures under different drug treatments^15^: **i**, disome average map in *M.pneumoniae* cells treated with RNA polymerase inhibitor pseudoridimycin (PUM); **j**, disome average in *M. pneumoniae* cells treated with the ribosome antibiotic spectinomycin (Spc). **k**, Overlay of Cm-treated and PUM-treated disome maps shows high similarity. **l**, Overlay of Cm-treated and Spc-treated disome maps.

In all three disome classes found in Cm-treated cells, ribosomal protein L9 of the leading ribosome adopted an extended conformation that reaches out to the following ribosome (Fig. 4a–c), similar to that found in the t–t disomes in untreated cells^15^ (Extended Data Fig. 6a–c). Disome class I is structurally similar to the t–t disomes in untreated *M.* *pneumoniae* cells, while classes II and III are more compact (Fig. 4a–c and Extended Data Fig. 6a–c). The most compacted disome class III was resolved to 8.7 Å, within which mRNA could be traced and modeled from the leading to the following ribosome, as well as the ribosomal proteins within the interface (Fig. 4d,e). Class III resembled the recently reported structures of in vitro collided ribosomes of both *B.* *subtilis*^27^ and *E.* *coli*^28^, in terms of the overall structure, mRNA trajectory and interface proteins (Extended Data Fig. 6d–g). Our class III disome maps, however, did not contain additional density that could be assigned to the ribosome rescue factors SmrB or MutS2 found in the in vitro collided disomes^27,28^, in line with *M.* *pneumoniae’*s genome lacking SmrB or other small MutS-related domain-containing proteins^17^. Other minor differences at the disome interface originate from *M.* *pneumoniae’s* specific set of amino acid extensions in proteins S5, S6 and S18, as well as the lack of protein S1 (refs. ^15,17^). Interestingly, we found such collided disomes to exist when reanalyzing data from *M.* *pneumoniae* cells treated with the RNA polymerase inhibitor pseudoridimycin, where leading ribosomes engaged in transcription–translation coupling are physically blocked by stalled polymerases, whereas this was not the case in cells treated with the antibiotic spectinomycin, which binds to the 30S neck and blocks translocation^15^ (Extended Data Fig. 6h–l and Extended Data Table 2).

Integrating the polysome analysis with the results from our classification of ribosome translation elongation intermediates showed the distribution of elongation states for the leading and following ribosomes to be related to the different disome configurations (Fig. 4a–c). Specifically, there was an enrichment of the rotated pretranslocational ‘A*, P/E’ state in the following ribosomes of the more compacted disome classes II and III. In contrast, the state distribution of either the leading or the following ribosomes in the less compact class I showed the same patterns as in monoribosomes (Fig. 4a and Extended Data Fig. 5e). While the resolutions of the disome maps were not sufficient to directly determine whether Cm was bound, considering the saturating concentrations of Cm applied (Methods) and the unambiguous identification of the Cm density in the vast majority of translation elongation intermediates (Fig. 3c), with the exception of the minor ‘A*, P/E’ class, we suggest that Cm is also bound in the majority of polysomes. In agreement with this, disome classes II and III were not present in untreated cells and appeared only upon Cm treatment. These results indicated that Cm induces ribosome collisions within polysomes and changes their functional organization in cells. Ribosome collisions could arise from a scenario wherein the leading ribosome is stalled by Cm on the arresting motifs but the following ribosome is still able to elongate. This is supported by the observation that the rotated pretranslocational intermediate ‘A*, P/E’ after peptidyl transfer was overrepresented in the following ribosomes of the two most compacted disome classes II and III, while it distributed evenly in the disome class I that is similar in arrangement to polysomes found in untreated cells.

## Discussion

In this work, we demonstrate that it is feasible to obtain maps with local resolutions better than 3 Å and, thus, atomic-level detail for large macromolecular complexes within intact cells. The use of a smaller pixel size and the acquisition of a larger dataset for Cm-treated *M.* *pneumoniae* cells in this study compared to our previous reports^14,15^ (1.33 Å and 137 cells versus 1.7 Å and 65 cells) enabled us to improve the resolution of the ribosome consensus map, while the overall *B* factor of 85 Å^2^ remained similar (Extended Data Fig. 1g). Our refined in-cell *M.* *pneumoniae* ribosome structure aligns with high-resolution in vitro structures from other bacteria^6,19,21,29,30^, down to the level of small cofactors and ions (Extended Data Fig. 1j–l). Nevertheless, the surprising finding of S4_LSU_ highlights the existence of different ribosome isoforms inside cells and underlines potential value in obtaining such structures toward exploring their functions; in addition to its canonical role in forming the mRNA entry site on the 30S subunit, S4 is known to have essential roles in suppressing translation initiation of mRNAs from its own α operon^31^, in the transcription antitermination complex during ribosome biogenesis^24,32^ and in guiding early 16S rRNA folding and 30S subunit assembly^33,34^. However, there has been no previous report on the involvement of S4 protein in 50S assembly. In light of the diverse roles of S4 in regulating RNA folding, we hypothesize that S4_LSU_ can serve as a chaperone in 50S folding and assembly. This function may be specific to mycoplasmas and S4_LSU_ may later dissociate over time, independent of translation. Alternatively, the additional association site on the LSU can serve as a buffering pool to regulate the available S4 proteins in cells. The S4_LSU_ binding site appears to be structurally conserved in many bacterial ribosomes and it may, therefore, serve common yet unknown functions across bacteria. This result demonstrates the unique potential of in-cell cryo-ET in the discovery of new association factors on large complexes.

Our in-cell structure revealed atomic details of how Cm interacts with the natural constituents of the ribosome PTC and sheds light on the molecular inhibition mechanism of Cm. In addition to the 23S rRNA nucleotides forming the main Cm-binding pocket, our structure supports the previously reported importance of a potassium ion that is coordinated by the pocket-forming nucleotides G2068 (G2061 in *E.* *coli*) and U2512 (U2504 in *E.* *coli*) (Fig. 2d). Dependence on K^+^ for Cm binding was first shown biochemically^35,36^ and then confirmed structurally^4,37^. Cm binding displaces two or more water molecules, which form the hydrogen bond network with the K^+^ and pivotal nucleotides in PTC in untreated ribosomes^30,37^. This explains resistance to Cm arising from rRNA mutations (for example, at position G2455 (G2447 in *E.* *coli*)) that do not directly interact with Cm but coordinate the K^+^ ion^38–41^ or from modification of Cm’s C3 hydroxyl group that is part of the K^+^ coordination network^4,42^.

Cm is long known to sterically prevent proper positioning of the aminoacyl moiety of the incoming aa-tRNA in the A-site of the PTC, leaving the nascent peptide connected to the P-site tRNA (Fig. 2). Our in-cell structure demonstrates that direct interaction between Cm and the nascent peptide, especially its residue at position −1, further stabilizes Cm’s occupation of the A-site, consistent with previous in vitro structures obtained with synthetic nonhydrolyzable peptide analogs^10,11^. Large side chains of this penultimate residue can clash with Cm, explaining why Ala and, to a lower extent, Ser and Thr are overrepresented in Cm-bound ribosomes^7^. Additionally, we demonstrated that the residue at position −3 is involved in forming the Cm pocket (Fig. 2d,e), which favors Lys to help seal the binding pocket and stabilize Cm binding^7^. Although Cm can bind to vacant ribosomes^4–6^, inhibition is not effective until the arresting nascent peptide motifs are synthesized to further stabilize Cm’s binding and enhance its blocking activity^7,8^. In its rotated conformation, 23S rRNA nucleotide A2069 (A2062 in *T.* *thermophilus* and *E.* *coli*) interacts with both Cm and the nascent peptide (Fig. 2d) and has an important role in sensing the nascent peptide and stabilizing Cm’s inhibition^6,10,43,44^. In untreated *M.* *pneumoniae* cells with active translation elongation, A2069 in the average map adopts the unrotated conformation^15^. Thus, Cm’s action goes beyond simple blocking of access of the aminoacyl moiety to the PTC; it reshapes the PTC by imposing changes to rRNA conformation, ion coordination network and the interaction with specific sequences of the elongating nascent peptide.

As a consequence of the impaired peptide bond formation, Cm also reshaped the functional landscape of ribosomes. We found more than 97% of cellular 70S ribosomes to be present in six different translation elongation states before peptidyl transfer, including EF-Tu•tRNA decoding and aa-tRNA accommodation intermediates (Fig. 3). The relative abundance for these states differed markedly from their distribution in native untreated cells^15^. Ribosomes with all three tRNAs bound (A, P and E) in the classical pretranslocational state before peptide bond formation were not detected at significant levels in untreated cells but account for 21% of all 70S in Cm-treated cells. This indicates a functional link between disassociation of the E-site tRNA and successful peptidyl transfer^45^. These findings further suggest that Cm’s inhibition of peptidyl transfer possibly results in repeated rounds of nonproductive accommodation and dissociation of aa-tRNA in the A-site, in agreement with a previous single-molecule fluorescence resonance energy transfer study^8^. Similarly, hygromycin A binding in a PTC region partially overlapping with Cm’s binding site leads to oscillation of the incoming aa-tRNA between the A/T-like and the accommodated positions^46^. Our data provide direct structural evidence for the occurrence of these intermediates upon antibiotic treatment in the native cellular context (Fig. 3). When peptide bond formation is inhibited or slowed down, increased dissociation of aa-tRNA can occur through the route used for kinetic proofreading^47^. Accordingly, futile rounds of ternary complex formation and guanosine triphosphate (GTP) hydrolysis on the ribosome can occur^48^. These unproductive cycles may contribute to diminishing the intracellular GTP pool. Cm and most ribosome-targeting antibiotics are bacteriostatic drugs that halt cell growth but do not kill bacteria. It was recently reported that *B.* *subtilis* uses (p)ppGpp-mediated cellular stress response to protect against Cm by lowering the intracellular GTP level and that increasing intracellular GTP levels enhances Cm lethality^49^. Therefore, on top of protein synthesis inhibition, Cm can turn ribosomes into nonproductive machines that consume energy, possibly contributing to an additional effect of the antibiotic on cellular physiology. It is possible that cells in turn adapt to such additional stress, relying on (p)ppGpp-mediated pathways to decrease the GTP level and suppress the futile accommodation cycles. A combination of PTC-targeting antibiotics with drugs suppressing protective bacterial cellular stress responses may represent a promising direction for future antibacterial treatment development.

Cm treatment further profoundly reorganized the translation machinery in cells, with a particular impact on polysome arrangements. We found that about 70% of the detected polysomes in Cm-treated cells represent disome classes that resemble in vitro collided disome structures^27,28^ but that do not exist in untreated cells^15^. Such collisions increase translation errors such as frameshifting^50,51^, which can be reduced by the ribosomal protein L9 (refs. ^51,52^). In native untreated cells, L9 of the leading ribosome interferes with elongation factor binding, especially EF-G, to the following ribosome, thereby mediating polysome coordination during active translation^15^. However, for polysomes in Cm-treated cells, where the leading ribosome is prolongedly stalled on arresting sequences, the following ribosome may be able to complete factor-independent translocation, albeit at lower efficiency^53,54^, and collide with the leading one. Indeed, it has been reported that Cm increases translation errors, especially frameshifting and nonsense suppression, but not misincorporation^55^. Hence, we suggest that Cm treatment induces ribosome collisions that contribute to Cm’s effect on the cellular level. As a general sensor and inducer of cellular stress responses, ribosome collisions caused by Cm may exert a more detrimental effect on cells than pure inhibition of protein synthesis^56–58^. Cells, on the other hand, may alleviate the effect of Cm through the cellular stress response and ribosome collision rescue mechanisms^56,59^. Mutations of the ribosome rescue genes in *B.* *subtilis*^27^ or *E.* *coli*^28^ lead to increased sensitivity to ribosome-targeting antibiotics including erythromycin and Cm, which have been reported to act in a context-dependent manner and, thus, induce ribosome collision, but not spectinomycin and hygromycin B, which do not exert such mechanisms. Although ribosome rescue proteins have yet to be annotated in *M.* *pneumoniae* and are possibly absent altogether because of its substantial genome reduction, our results suggest a general mechanism for antibiotics that act in a sequence-dependent manner to additionally induce ribosome collisions as part of their cellular mechanisms of action.

To conclude, our study provides a comprehensive understanding of Cm’s inhibitory effect at the atomic, molecular and cellular levels and complements the current context-dependent inhibition model of Cm and other PTC-targeting antibiotics. We demonstrate that the context-dependent action of Cm is not only reflected in ribosome stalling on specific nascent peptide sequences but also propagates to the cellular scale in the native context of polysomes and possibly associated cellular stress response pathways. This work establishes how emerging in-cell structural biology approaches can advance mechanistic understanding of drug action in their natural context.

## Methods

### Cryo-ET data collection and processing

*M.* *pneumoniae* cell cultivation on grids and sample preparation were performed as previously described^15,60^. Cm (Sigma-Aldrich, C0378) was added to the cell culture in the fast-growing phase at a final concentration of 0.2 mg ml^−1^ and incubated for about 15 min before plunge-freezing. Cryo-ET data collection was performed on a Titan Krios G3i microscope equipped with a Gatan energy filter and K3 camera using SerialEM (version 3.9)^61^. Tilt series were collected using a dose-symmetric scheme^62^, with the following settings: magnification, ×64,000; pixel size on sample, 1.329 Å; tilt range, −60° to 60° with 3° interval; energy filter slit, 20 eV; K3 camera in noncorrelated double sampling counting mode; target dose rate on camera, 20 e^−^ per pixel per second; ten frames per tilt image; constant exposure time for each tilt; total dose, 137 e^−^ per Å^2^. For different tilt series, the target defocus values ranged from 1 to 3.25 μm. In total, 139 tilt series were used for data processing and analysis.

### Image processing and ribosome structure refinement

Cryo-ET data were processed in Warp (version 1.0.9)^14^, including frame motion correction, contrast transfer function (CTF) estimation and tilt series sorting. Tilt series alignment was performed in etomo (version 4.11)^63^ using gold fiducials. Tomograms were first reconstructed at 6× binning (voxel size = 7.974 Å) in Warp, after importing the etomo alignments. Ribosome picking was performed using template matching in pyTOM (version 0.9.7.1)^64^ and the top 600 or 900 ranking cross-correlation hits for every tomogram were extracted depending on the cellular coverage area. In total, 51,783 subtomograms were extracted from 139 cellular tomograms at 4× binning (voxel size = 5.316 Å) in Warp and subjected to 3D classification to remove false positives and free 50S in RELION (version 3.0.8)^65^. Two of the 139 tomograms contained fewer than 20 good particles after classification. They were excluded from subsequent processing because too few particles can lead to suboptimal performance of multiparticle refinement. In total, 30,774 subtomograms of 70S ribosomes were extracted from 137 tomograms at smaller voxel sizes for the following refinement and classification.

Initial refinement (alignment and averaging) of the 70S ribosome subtomograms was performed in RELION (version 3.0.8). The alignment parameters for all particles and the average map were imported into M (version 1.0.9) for multiparticle refinement^14^. Structure-based refinement of both geometric (image and volume warping, particle poses and tilt series alignments) and CTF parameters was performed for five rounds in M, which also automatically updates the metadata files in the associated Warp processing folders. After M refinement on the 70S ribosomes with a global mask, focused refinements with 30S and 50S masks were performed simultaneously with only the ‘particle pose’ option to improve local map quality. Fourier shell correlation (FSC) calculation between randomly split half subsets, local resolution estimation and additional postprocessing were performed in M and RELION.

### Atomic model building of high-resolution ribosome averages

The atomic models of the *M.* *pneumoniae* 30S ribosomal subunit (PDB 7OOC) and 50S ribosomal subunit (PDB 7OOD)^15^ were initially docked into the density maps with UCSF Chimera^66^ and manually adjusted in Coot^67^. To model regions of different local resolutions, such as intrinsically flexible RNA or the N-terminal parts of the nascent peptide, different *B* factors (−10 to +120) were used to sharpen or blur the density map in RELION postprocessing^65^. As the modeled mRNA represents an average of all native *M.* *pneumoniae* mRNAs bound to the imaged ribosomes, a random-sense mRNA sequence was chosen, except for the three codons, which were adjusted to the anticodons of chosen tRNAs. The tRNAs were selected on the basis of the prevalence of amino acids found at positions +1, 0 and −1 of the nascent chains in *E.* *coli* ribosomes upon Cm treatment^7^. Here, position +1 corresponds to the A-site amino acid, position 0 corresponds to the P-site or ultimate amino acid and position −1 corresponds to the penultimate amino acid of the nascent chain, respectively. A poly(Ala_(<__−__4)_-Lys_(__−__3)_-Ala_(__−__2)_-Ala_(−1)_-Asp_(0)_) polypeptide was modeled as the nascent chain (the poly(Ala) part is labeled as poly(UNK) in the deposited model, as the identity is unknown). The tRNA sequences and the codon usage were adjusted to match the experimentally determined most frequently used ones in *M.* *pneumoniae*^68^, namely, deacylated tRNA^Ala^ (gene cluster, trnD; gene name, *MPNt01*; anticodon, UGC; codon, GCC; U•C wobble pair) for the E-site (where present), nascent chain-acylated tRNA^Asp^ (cluster, trnB; gene, *MPNt11*; anticodon, GUC; codon, GAU; G•U wobble pair) for the P-site and acylated Lys-tRNA^Lys^ (cluster, trnE; gene, *MPNt28*; anticodon, CUU; codon, AAG) for the A-site, respectively. To ensure correct placement and refinement of the acylated CCA tail of the A-site tRNA, lysinyl-adenosine monophosphate was created (AK9) in Coot and restraints were generated using the module ‘eLBOW’ (ref. ^69^) in PHENIX^70^. AK9 was temporarily added to the 3′ end of tRNA-Lys, replacing the adenosine at position 76. Later, because of wwPDB’s ‘nucleotide + amino acid’ definition, AK9 was set back to a single nucleotide and amino acid linked to a standard adenosine. The starting model for the N-terminal ribosome-binding domain (residues 29–58) of the trigger factor from *M.* *pneumoniae* (AF-P75454), identified on the basis of its position near the peptide exit site (Extended Data Fig. 2a–c), was retrieved from the AlphaFold database (https://alphafold.ebi.ac.uk/)^71^. All models were refined over multiple rounds using the module ‘phenix.real_space_refine’ in PHENIX and interactive model building and refinement in Coot, using libG restraints^72^ for the RNA. The quality of all refined models was assessed using the ‘comprehensive model validation’ function in PHENIX and wwPDB validation server (https://validate.wwpdb.org). The model validation statistics in Tables 1 and 2 were calculated using MolProbity^73^. The rRNA secondary-structure representation from the PDB file was performed with RNApdbee 2.0 software^74^ and the image was produced with VARNA^75^.

### Protein identification and atomic model building for ribosomes with S4_LSU_

Ribosomes showing an additional density near 23S rRNA helices 16–18 were sorted out through focused classification with a spherical mask covering the additional density (Extended Data Fig. 2a). Parallel RELION jobs were performed to mitigate variations. After obtaining the density map, we aimed to identify the protein on the basis of its fold. As automated building of α-helices and β-strands into the additional density did not yield a meaningful model, several poly(Ala) stretches were placed manually in Coot with the ‘place helix/strand here’ module. Because of the visibility of a few side chains in the density, the directionality of the potential α-helices could be deduced. Additionally, some parts of potential β-strands were built and all identified polypeptide stretches were connected through random coil stretches to yield a single polypeptide of 123 residues. The model was submitted for a 3D structure similarity search to the Dali server (http://ekhidna2.biocenter.helsinki.fi/dali/)^76^ against the PDB. This search yielded PDB 5WNU (chain D)^77^ as the top hit (*z* score = 11.2, root-mean-square deviation (r.m.s.d.) = 2.4 Å, lali = 112), which corresponds to 30S ribosomal protein S4 from *T.* *thermophilus* (UniProt P80373). On the basis of the search result, we placed a second copy of *M.* *pneumoniae* 30S ribosomal protein S4 (now called S4_LSU_), N-terminally truncated to residues 47–205, into the additional density, which gave a robust fitting that only required minor adjustment (Extended Data Fig. 2d). The N-terminal 46 residues were built de novo into the density in Coot.

### Classification of translation elongation intermediates

The 3D classification of the 70S ribosomes was performed in RELION (version 3.0.8) using the subtomograms re-extracted in M (version 1.0.9) after multiparticle refinement. The hierarchical classification strategy and procedure are similar to those described in our previous study^15^ and are illustrated in detail in Extended Data Fig. 4a. At least three tiers of RELION classification jobs were performed. First, the 70S ribosomes were classified using a global 70S mask to remove false positives or ‘bad’ particles. Second, ribosomes were sorted according to the different tRNA binding states, using either a focused mask covering the SSU plus all possible translational factor binding regions or a spherical mask covering the A, P and E tRNA-binding sites. Third, classification was based on the different elongation factor and A/T tRNA-binding states, using a spherical mask focusing on that region. A total of 15,332 ribosomes with clear A-site and P-site tRNAs were first separated and further classified on the basis of the E-site tRNA density into ‘A, P’ (8,854 particles) and ‘A, P, E’ (6,478 particles). The remainder were classified into five major groups: 7,048 ribosomes with EF-Tu•tRNA and P-site tRNA, 5,661 ribosomes with partially resolved A-site tRNA (only the tRNA tip close to the decoding center is well resolved; labeled as ‘a’) and P-site tRNA, 787 ribosomes with A-site tRNA and hybrid P/E-tRNA, 769 ribosomes with less resolved density near the P-site and 1,177 ribosomes with a dim 30S subunit. The last two classes did not result in any interpretable density maps and were, thus, not further analyzed. The 7,048 ribosomes with EF-Tu•tRNA and P-site tRNA and the 5,661 ribosomes with partially resolved A-site tRNA were finally classified on the basis of the E-site tRNA states. In each tier, at least three parallel classification jobs (with identical or slightly different settings or with different masks) were performed and the most consistent job was selected to sort the particles. For each particle classification and sorting, follow-up classification runs were performed until no new or different subclasses emerged.

### Spatial and statistical analysis of polysomes

The polysome annotation procedure was performed as described before using functions in TOM toolbox^15^, which considers both the relative positions and the orientations of neighboring ribosomes in the 3D cellular volume. Polysomes in this study only refer to closely assembled ribosomes with a distance threshold of 7 nm (ref. ^15^). The distance determined from a manually defined mRNA exit site on the preceding ribosomes (*i*) to the mRNA entry site of the following ribosomes (*i* + 1) was used to determine whether the two ribosomes belong to the same polysome. In total, 4,838 ribosomes were annotated to be within polysomes. Additionally, neighboring ribosome–ribosome pairs within the polysomes were sorted on the basis of their relative rotations^15,26^: 2,406 t–t pairs with an mRNA exit-to-entry distance of 3.3 ± 1.5 nm and 227 top–bottom pairs with a distance of 5.4 ± 1.1 nm.

Disomes (ribosome pairs within polysomes) were additionally structurally classified by first extracting the 4,838 annotated ribosomes with a large box (3.1 Å per voxel, box size = 256 voxels) and applying focused classification with a spherical mask covering the position of the following ribosome. Three disome structures (disome class I, 654; class II, 387; class III, 963) were classified in RELION. The remaining particles in classes that did not generate meaningful densities were not used for further analysis. The functional states of the leading and the following ribosomes within the disomes were mapped by integrating the above ribosome classification results. Similar classification was performed for disomes in the pseudouridimycin-treated and spectinomycin-treated datasets generated in our previous study^15^.

### Modeling of compacted disome structure

Modeling of the most compacted disome class III (map global resolution of 8.7 Å at FSC = 0.143) was performed using the built 30S and 50S subunits as the starting models, which were first rigid-body fitted into the density map using Chimera and Coot. Several parts of the models for the leading (*i*) and following (*i* + 1) ribosomes required adjustments in Coot to be accommodated in the disome map. For example, residues Arg121–Ala137 of protein S6_*i*_ must fold differently (compared to free 70S) to avoid clashes with protein S5_*i*+1_. The C terminus of protein S6_*i*+1_ detaches from the ribosome and is disordered from residue Ser168 onward, as it would clash with 70S_*i*_. The CTD of protein L9_*i*_ is in an extended conformation and blocks the translation factor binding site of the following 70S_*i*+1_. The A-site of 70S_*i*+1_ is empty, as the ternary complex cannot form. The A-site finger of the following ribosome was found in two conformations and the monitoring bases (16S_*i*+1_ rRNA bases 1467–1468) are mainly in the ‘flipped-in’ conformation. We also used information from in-cell crosslinking mass spectrometry^60^ to guide model building where the path of the polypeptide was somewhat unclear, mainly for the flexible termini of some ribosomal proteins. The model for mRNA located in between the two ribosomes was built to accommodate exactly ten codons (30 nucleotides) between the two A-sites, similar to other structures of collided bacterial disomes^27,28^ and matching biochemical studies^51^, ranging from eight to ten codons.

Structural visualization and production of illustrations were performed in Chimera^66^ and ChimeraX^78^. Statistical analysis and plotting were performed in MATLAB 2016b.

### Reporting summary

Further information on research design is available in the Nature Portfolio Reporting Summary linked to this article.

## Online content

Any methods, additional references, Nature Portfolio reporting summaries, source data, extended data, supplementary information, acknowledgements, peer review information; details of author contributions and competing interests; and statements of data and code availability are available at 10.1038/s41594-024-01441-0.

## Supplementary information

Reporting Summary

## Source data

Source Data Fig. 3 Statistical source data.

Source Data Fig. 4 Statistical source data.

Source Data Extended Data Fig. 1 Statistical source data.

Source Data Extended Data Fig. 2 Statistical source data.

Source Data Extended Data Fig. 4 Statistical source data.

Source Data Extended Data Fig. 5 Statistical source data.

## Figures and Tables

**Extended Data Table 1:**
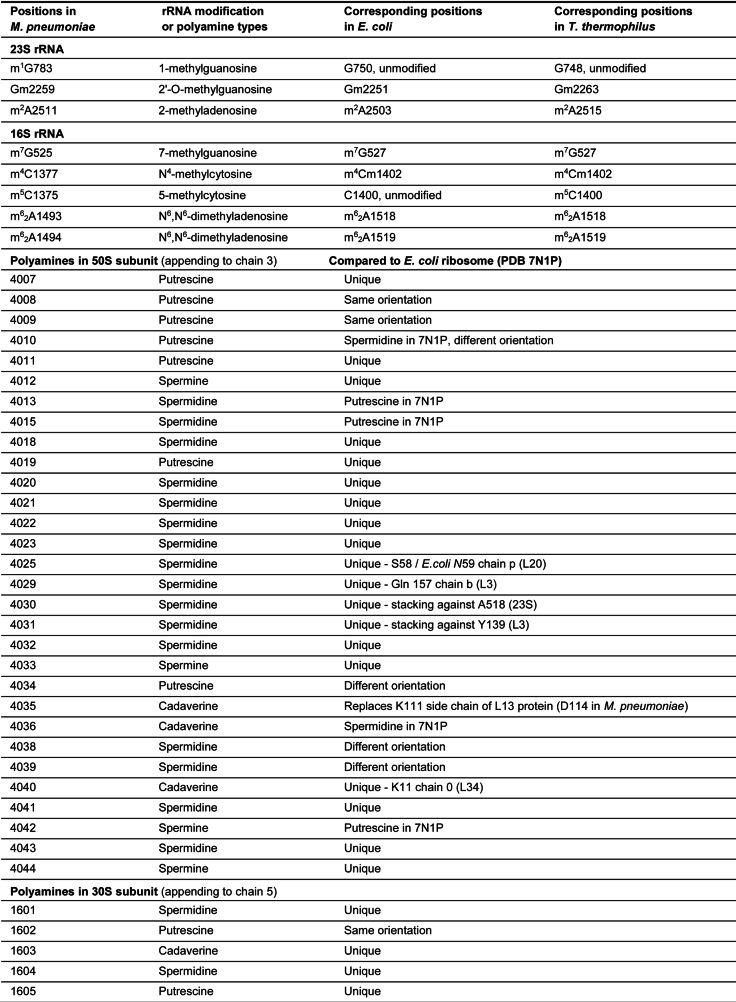
Modeled rRNA modifications and polyamines in *M. pneumoniae*

**Extended Data Table 2:**
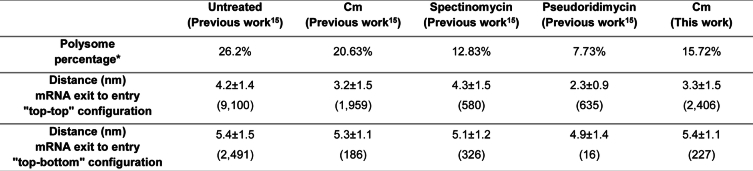
Polysome statistics in native untreated and antibiotic-treated *M. pneumoniae* cells

Footnote: *Ribosomes that are spatially annotated as polysomes out of all 70S ribosomes. The mean and standard deviation for distances from the mRNA exit site of the leading ribosome to the mRNA entry site of the following ribosome for pairs with ‘top-top’ and ‘top-back’ configurations as defined previously^26^ are displayed in nanometers. The numbers of ribosome pairs are provided below in parentheses.

## Data Availability

Detailed information for all maps and models generated in this work is provided in Tables 1 and 2. The raw cryo-ET data were deposited to the EM Public Image Archive under accession code EMPIAR-11520. Maps were deposited to the EM Data Bank under accession codes EMD-17132, EMD-17133, EMD-17134, EMD-17135, EMD-17136, EMD-17137, EMD-17138, EMD-17139, EMD-17140, EMD-17141, EMD-17142, EMD-17143, EMD-17144, EMD-17145, EMD-17146 and EMD-17147. Atomic models were deposited to the PDB under accession codes 8P6P, 8P8B, 8P7X, 8P7Y, 8P8W and 8P8V. Maps and atomic models used from previous studies were obtained from the PDB (7OOC, 7OOD, 7P6Z, 5WNU, 6QNR and 7N1P). The predicated model of the trigger factor was obtained from the AlphaFold Protein Structure Database (AF-P75454). The *M.* *pneumoniae* M129 protein and RNA sequences were obtained from the National Center for Biotechnology Information (NC_000912.1). Source data are provided with this paper.
