# Supplementary material for: Structural insights into context-dependent inhibitory mechanisms of chloramphenicol in cells
Source: Nat Struct Mol Biol. 2024 Dec 12;32(2):257–67. doi: 10.1038/s41594-024-01441-0 (PMC11832420; doi:10.1038/s41594-024-01441-0)
Supplement: Supplementary file 1 — Reporting Summary [file 41594_2024_1441_MOESM1_ESM.pdf]

nature research

Corresponding author(s): Julia Mahamid

# Reporting Summary

Nature Research wishes to improve the reproducibility of the work that we publish. This form provides structure for consistency and transparency in reporting. For further information on Nature Research policies, see Authors & Referees and the Editorial Policy Checklist.

## Statistical parameters

When statistical analyses are reported, confirm that the following items are present in the relevant location (e.g. figure legend, table legend, main text, or Methods section).

| n/a | Confirmed | |
|---|---|---|
| ☐ | ☒ | The exact sample size ($n$) for each experimental group/condition, given as a discrete number and unit of measurement |
| ☐ | ☒ | An indication of whether measurements were taken from distinct samples or whether the same sample was measured repeatedly |
| ☐ | ☒ | The statistical test(s) used AND whether they are one- or two-sided<br>*Only common tests should be described solely by name; describe more complex techniques in the Methods section.* |
| ☒ | ☐ | A description of all covariates tested |
| ☐ | ☒ | A description of any assumptions or corrections, such as tests of normality and adjustment for multiple comparisons |
| ☐ | ☒ | A full description of the statistics including central tendency (e.g. means) or other basic estimates (e.g. regression coefficient) AND variation (e.g. standard deviation) or associated estimates of uncertainty (e.g. confidence intervals) |
| ☐ | ☒ | For null hypothesis testing, the test statistic (e.g. $F$, $t$, $r$) with confidence intervals, effect sizes, degrees of freedom and $P$ value noted<br>*Give P values as exact values whenever suitable.* |
| ☒ | ☐ | For Bayesian analysis, information on the choice of priors and Markov chain Monte Carlo settings |
| ☐ | ☒ | For hierarchical and complex designs, identification of the appropriate level for tests and full reporting of outcomes |
| ☒ | ☐ | Estimates of effect sizes (e.g. Cohen's $d$, Pearson's $r$), indicating how they were calculated |
| ☐ | ☒ | Clearly defined error bars<br>*State explicitly what error bars represent (e.g. SD, SE, CI)* |

*Our web collection on statistics for biologists may be useful.*

## Software and code

Policy information about availability of computer code

| | |
|---|---|
| Data collection | SerialEM 3.9, doi: 10.1016/j.jsb.2005.07.007 |

| | |
|---|---|
| Data analysis | IMOD 4.9.4, doi: 10.1006/jsbi.1996.0013;<br>Warp 1.0.9, doi: 10.1038/s41592-019-0580-y;<br>MATLAB 2016b & 2019b (https://www.mathworks.com);<br>TOM matlab toolbox release-2008, doi: 10.1016/j.jsb.2004.10.006;<br>RELION 3.0.7 & 3.0.8, doi: 10.7554/eLife.42166;<br>M 1.0.9, doi: 10.1038/s41592-020-01054-7;<br>Coot 0.9 or later, doi: 10.1107/S0907444910007493;<br>Phenix 1.18, doi: 10.1107/S2059798319011471;<br>Chimera 1.13.1, doi: 10.1002/jcc.20084;<br>ChimeraX 1.1.4, doi: 10.1002/pro.3943;<br>DALI server (doi: 10.1093/nar/gkq366): http://ekhidna2.biocenter.helsinki.fi/dali/, accession time August 2022;<br>RNApdbee version 2.0: doi: 10.1093/nar/gky314;<br>AlphaFold Protein Structure Database (doi: 10.1038/s41586-021-03819-2): https://alphafold.ebi.ac.uk/;<br>wwPDB validation server (doi: 10.1038/nsb1203-980): https://validate.wwpdb.org;<br>MolProbity 4.5, doi: 10.1002/pro.3330;<br>MATLAB script for polysome annotation (doi: 10.1038/s41586-022-05255-2): GitHub repository https://github.com/xueliang4906/polysome_detect |

For manuscripts utilizing custom algorithms or software that are central to the research but not yet described in published literature, software must be made available to editors/reviewers upon request. We strongly encourage code deposition in a community repository (e.g. GitHub). See the Nature Research guidelines for submitting code & software for further information.

## Data

Policy information about availability of data

All manuscripts must include a data availability statement. This statement should provide the following information, where applicable:

- Accession codes, unique identifiers, or web links for publicly available datasets
- A list of figures that have associated raw data
- A description of any restrictions on data availability

Detailed information for all maps and models generated in this work is provided in Tables 1 and 2. The raw cryo-ET data are deposited in the Electron Microscopy Public Image Archive (EMPIAR) under accession code EMPIAR-11520. Maps are deposited in the Electron Microscopy Data Bank (EMDB) under accession codes: 17132, 17133, 17134, 17135, 17136, 17137, 17138, 17139, 17140, 17141, 17142, 17143, 17144, 17145, 17146, 17147. Atomic models are deposited in the Protein Data Bank (PDB) under accession codes: 8P6P, 8P8B, 8P7X, 8P7Y, 8P8W, 8P8V. Maps and atomic models used from previous studies were obtained from the PDB (https://www.rcsb.org/structure/7OOC, https://www.rcsb.org/structure/7OOD, https://www.rcsb.org/structure/7P6Z, https://www.rcsb.org/structure/5WNU, https://www.rcsb.org/structure/6QNR, https://www.rcsb.org/structure/7N1P). The Mycoplasma pneumoniae M129 protein and RNA sequences are from NCBI Reference Sequence NC_000912.1(https://www.ncbi.nlm.nih.gov/nuccore/NC_000912.1). The predicated model of trigger factor is from AlphaFold 2 database (https://alphafold.ebi.ac.uk/AF-P75454). Source data are provided for quantification and plots presented in Figures 3b, 4a, Extended Data Figures 1d, 1g, 1h, 1i, 2l, 2m, 4j, 5c, 5d, 5e, 5i.

# Field-specific reporting

Please select the best fit for your research. If you are not sure, read the appropriate sections before making your selection.

☒ Life sciences ☐ Behavioural & social sciences ☐ Ecological, evolutionary & environmental sciences

For a reference copy of the document with all sections, see nature.com/authors/policies/ReportingSummary-flat.pdf

# Life sciences study design

All studies must disclose on these points even when the disclosure is negative.

| | |
|---|---|
| Sample size | No sample size calculation was performed. The sample size was considered to be sufficient based on the refinement results, where resolution achieved the physical limit of the data. For cryo-ET data collection, at least 3 grids were screened, and 1 of these was used for final data collection based on the ice conditions. In one grid, there are at least a few hundreds of cells that can be used for data collection, and 142 of these were selected for final data collection based on the suitability of ice thickness. The sample size was considered sufficient as the obtained maps were resolved to the pixel size limit of this data (Nyquist resolution). After extensive classification, most classes contain more than one thousand particles, which are expected to ensure reliable classification results in RELION (DOI: 10.1016/bs.mie.2016.04.012). These factors indicate that the current data size is sufficient for the structural and computational analysis presented here. |
| Data exclusions | For cryo-ET data collection, grids with thick ice or severe deformation were discarded. Cells that are clustered in thick ice areas were excluded, as these cells do not contribute high-quality data for the subsequent structural analysis.<br>For data processing, tilt-series that contained ice contaminants or more than 4 tilt images with failed tracking were excluded from the final analysis. Such data represented 3 out of the 142 imaged cells. Two of the remaining 139 tomograms contained less than 20 good ribosomes after initial RELION classification and were not included in M processing as too few particles cause suboptimal refinement. Finally, 137 cells were used for the analysis presented in this work. |
| Replication | For cryo-ET sample preparation and data collection, at least 3 grids were prepared for the experimental conditions and cryo-ET data were collected on the grid with the best ice thickness. All computational experiments were repeated to find the best parameters and reproduce the results. Once all parameters were optimized, replication attempts were successful and the results were reproducible: For refinements in |

RELION or M, jobs were repeated to ensure the same resolution can be achieved. The refinement and post-processing (resolution determination) followed the "gold standard" in the cryo-EM/ET field, i.e. the data is randomly split into to half sets and the half sets were refined independently. The reported resolutions are based on FSC at 0.143 calculated between densities produced from the independent half sets. For classification, more than 3 parallel jobs were performed to mitigate variations associated with single jobs and to ensure the classification is exhaustive. More follow-up classification jobs were performed to confirm the convergence of the previous classifications. For all structural modeling, at least 2 rounds of refinements were performed, followed by validation and visual inspection to confirm the model quality.

| | |
|---|---|
| Randomization | No randomization was performed for cryo-ET sample preparation and data collection. The selection of grids/cells for data collection was based on ice thickness, cell positioning within the film hole, fiducial markers distribution, etc. For cells in regions meeting standards that are required for high-quality cryo-ET data, the subsequent processing was performed without considerations of cell shapes or other visible features. For structure refinement in M or RELION, particles were randomly divided into two half datasets by the software. For classification, particles are first randomly divided evenly into classes by RELION. For bioinformatics analysis, structural modeling, and polysome annotation, randomization is not relevant because all were performed according to the confirmed sequences, maps and coordinates. |
| Blinding | No blinding to group allocation was performed during data collection and initial processing: the consensus refinement showed clear density for Cm, which could not be assigned without knowledge of the protein and RNA sequences used for building the model, as well as the identity of the small molecule. Nevertheless, the coordination of Cm in this study is different from previous published structures and emerged from the data. Non-supervised classification resulted in 6 classes, all binding the small molecule, which could not be predicted prior to the analysis. Nevetheless, knowledge on the identity of the molecule and its binding cite were essential for the interpretation of the data. |

# Reporting for specific materials, systems and methods

## Materials & experimental systems

| n/a | Involved in the study |
|---|---|
| ☒ | ☐ Unique biological materials |
| ☒ | ☐ Antibodies |
| ☒ | ☐ Eukaryotic cell lines |
| ☒ | ☐ Palaeontology |
| ☒ | ☐ Animals and other organisms |
| ☒ | ☐ Human research participants |

## Methods

| n/a | Involved in the study |
|---|---|
| ☒ | ☐ ChIP-seq |
| ☒ | ☐ Flow cytometry |
| ☒ | ☐ MRI-based neuroimaging |
